# Behavior of PNIPAM Microgels in Different Organic Solvents

**DOI:** 10.3390/molecules27238549

**Published:** 2022-12-05

**Authors:** Galina A. Komarova, Elena Yu. Kozhunova, Igor I. Potemkin

**Affiliations:** 1 Physics Department, Lomonosov Moscow State University, Leninskie Gory 1-2, Moscow 119991, Russia; 2 A.N. Nesmeyanov Institute of Organoelement Compounds, Russian Academy of Sciences, Vavilova St. 28, Moscow 119991, Russia

**Keywords:** microgel, thermosensitive, stimuli-responsive, poly(*N*-isopropylacrylamide), organic solvent, oil, solubility, water insoluble, Sudan III

## Abstract

In this research, we studied, in detail, the behavior of common PNIPAM microgels, obtained through surfactant-free precipitation polymerization, in a number of organic solvents. We showed that many of the selected solvents serve as good solvents for the PNIPAM microgels and that the size and architecture of the microgels depend on the solvent chosen. Expanding the range of solvents used for PNIPAM microgel incubation greatly enhances the possible routes for microparticle functionalization and modification, as well as the encapsulation of water-insoluble species. In this demonstration, we successfully encapsulated water-insoluble Sudan III dye in PNIPAM microgels and prepared the aqueous dispersions of such composite-colored microparticles.

## 1. Introduction

Microgels are swollen polymer gel particles forming colloidally stable dispersions when dissolved in a solution [1]. Typically, the size of the microgel particles is between 10 and 1000 nm. The microgels can exhibit a response to external stimuli, such as temperature, pH, flow, magnetic and electric fields, osmotic pressure, or light [2,3,4,5,6,7,8]. In many cases, microgel functionality is based on the chemical composition of the polymer network. It can be modified during microgel synthesis or by soaking compounds into a polymer network during a swelling process of dry microgels [2]. There are many applications for microgels in different areas, such as optics, coating, medicine, agriculture, actuators, etc. [9,10,11,12,13,14,15,16,17]. Most of the reports dedicated to microgels prepared via the self-assembly mechanism observed in precipitation polymerization used poly(*N*-isopropylacrylamide) (PNIPAM) as a main component [6,18,19,20,21,22]. PNIPAM-based microgels are of interest for different applications due to the tunability of their chemical and physical properties. For example, PNIPAM microgels modified with aminophenylboronic acid show glucose-responsive properties at a certain temperature, pH, and ionic strength [23,24,25]. A composite PNIPAM and Calcon dye microgel shows both pH and H_2_O responses [26]. Interpenetrating network microgels based on PNIPAM and acrylic acid show both thermo- and pH-responsive properties, forming microphase-separated morphologies in certain conditions [27,28,29]. Optical sensors for environmental humidity have been constructed from poly(*N*-isopropylacrylamide-co-acrylic) acid [30]. Kim et al. demonstrated that poly(*N*-isopropylacrylamide-*co*-acrylic acid) (PNIPAM-AAc) microgels can be used as tunable self-assembled microlenses [31].

Usually, water is used as a solvent for PNIPAM microparticles—both for research and applications. There are almost no data on the behavior of these species in organic solvents, except for research on one chosen medium [32,33]. In studies [34,35], the properties of linear PNIPAM and PNIPAM macrogels soaked in organic solvents were described. Yet, organic solvents are a powerful tool for the further modification of prepared microgels [36]. For example, an esterification reaction would allow for attachment to common PNIPAM-AAc microgels with a number of functional groups [37]. The loading of water-insoluble active compounds to PNIPAM microparticles is of importance for a variety of applications. This process is facilitated by the use of non-aqueous solutions. It is known that PNIPAM microgels are amphiphilic in nature due to the presence of hydrophobic and hydrophilic groups in each monomer unit. Kawaguchi et al. were the first to report that PNIPAM microgel particles can absorb hydrophobic species. They investigated the temperature dependence of human gamma-globulin absorption by PNIPAM microgels [38]. Azobenzene-containing microgels were used to fabricate optical materials whose properties depend on the wavelength of light irradiation [39]. Semiconductor quantum dots were loaded into thermo-responsive microgels via reversible transfer from organic solvents to water [40].

A number of applications uses organic liquids as a main operating solvent, such as non-aqueous redox-flow batteries and solid-state batteries. Crosslinked microparticles are a promising material for such systems [41,42,43]. Microgels can be implemented as stimuli-responsive recyclable catalytic systems for organic synthesis and serve as nanoreactors [44]. For example, in study [32], the horseradish peroxidase enzyme was immobilized in PNIPAM microgels through the solvent exchange from water to isopropanol. The composite particles showed enhanced specific activity of the biocatalyst. The proposed general method allows for the transfer of a water-soluble enzyme to an organic phase, reaching high catalytic activity.

Thereby, in this research, we present practical information about the behavior of PNIPAM microgels in a number of commonly used organic solvents at room temperature. Colloidal dispersions of PNIPAM microgels are widely used by many researchers and engineers; thus, extending our knowledge of linear PNIPAM and macrogels to the case of spherical microparticles with an uneven polymer density distribution and abundant hydrophilic initiators is of importance. We show the dependence of the PNIPAM microgel hydrodynamic radius *R*_h_, radius of gyration *R*_g_, and shape factor *R*_g_*/R*_h_ on the type of solvent at 23 °C. Further, we explore the encapsulation of a hydrophobic Sudan III dye [1-(4-(phenyldiazenyl)phenyl]azonaphthalene-2-ol] into the PNIPAM microgel through soaking dry particles in colored chloroform. We were the first to demonstrate the ability of such Sudan III-loaded microgels to form a stable aqueous dispersion. Sudan III dye is a strongly lipophilic diazo dye. It has long been used as a colorant for fats, oils, textile, waxes, and other hydrophobic materials [45]. Sudan III dyes, as well as other azo dyes, are water pollutants due to their widespread use in industries [46,47].

## 2. Results and Discussion

### 2.1. Swelling of Microgels in Different Solvents

For this investigation, we chose a number of organic solvents frequently used either as a medium for chemical modification and the loading of polymer particles or as a part of the functional material. A list of the solvents is presented in Table 1. We dissolved a small amount of PNIPAM microgels in the listed chemicals and evaluated the solubility of the particles at 23 °C. In cases where no precipitate was detected, we measured the hydrodynamic radius *R_h_* and the radius of gyration *R*_g_ of the microgels (see Table 1). Moreover, the shape factor *R*_g_*/R*_h_ and the ratio between the hydrodynamic radii in a solvent and in water *R*_h_*/R*_h(H_2_O)_ were calculated. It should be noted that the behavior of the microgel could not be evaluated in toluene and benzene due to the close proximity of the refractive indexes of these solvents and the NIPAM polymer. Examples of the intensity correlation functions and the angular dependencies of the relaxation time of microgel solutions are presented in Figures S3 and S4.

We found that microgels form stable colloid solutions and swell in water-miscible polar organic solvents, such as dioxane and tetrahydrofuran, at room temperature. Moreover, PNIPAM particles swelled in alcohols, including poorly water-soluble fatty alcohols. The shape factor *R*_g_*/R*_h_ of the particles was found to be lower than 0.5, indicating a “denser core—looser corona” structure. This structure points to the absence of a compact outer layer and promotes the accessibility of many polymer units comprising the microparticle. In some good solvents, the *R_h_* of the microgels can be bigger or smaller than that in water. This should be kept in mind when planning size-sensitive applications.

A phase transition of the PNIPAM gel caused by a collapse of its subchains upon reaching the lower critical solution temperature of 32 °C is characteristic only for the case of a PNIPAM gel being placed in pure water. For other solvents, the conditions of such transitions would be significantly different or the phase transition may even be absent.

For procedures requiring the loading of active hydrophobic species, the solvents immiscible with water are of importance. In non-polar solvents, such as cyclohexane and various oils, for example, tetradecane, the PNIPAM microgels precipitate. We found only a few water-immiscible (or nearly immiscible) solvents that are good for PNIPAM microgels, namely chloroform and the fatty alcohols 1-octanol and 1-butanol. The formation of hydrogen bonds plays a role in the solubility of PNIPAM in chloroform [35,48].

Fatty alcohols, such as 1-octanol, interact with the hydrophobic (isopropyl) side group carried by each monomer in PNIPAM and establish hydrogen bonds with its amide groups, modifying its amphiphilic qualities [49]. It is probable that some alcohols with a higher number of carbon atoms could also serve as a good solvent for PNIPAM microgels. PNIPAM microgels did not dissolve in mineral oil, hexane, decane, tetradecane, and cyclohexane.

It is of interest that while homogeneous macrogels usually retain their spatial density distribution, regardless of the solvent used, PNIPAM microgels, prepared via the precipitation polymerization method, tend to change their radial density distribution depending on the solvent type. Among the reasons for that effect is the non-uniform crosslinking density distribution and the presence of dangling end chains [1,19]. For example, in acetonitrile, the PNIPAM microgel particle appears to be an almost homogeneous sphere (*R_g_/R_h_* ratio is equal to 0.88 ± 0.06, see Table 1), while in *t-*butanol, the microgel structure transforms into a strongly pronounced denser-core looser-corona structure (*R_g_/R_h_* ratio is equal to 0.34 ± 0.04). Thus, not only the overall size and density of the microgel change, but also its architecture does. It is known, for example, that the ratio between the microgel corona and core sizes has a significant effect on the particle arrangement on the liquid–liquid interface [50].

**Table 1:** Characteristics of solvent solubility parameters (*δ*_sol._) [51,52,53] and sizes of PNIPAM microgels in different solvents at 23 °C.

Name	*δ*_sol._, cal^1/2^ cm^−3/2^	*R*_h_, nm	*R*_g_, nm	*R*_g_/*R*_h_	*R*_h_/*R*_h(H_2_O)_
tetrahydrofuran	9.1	273 ± 6	161 ± 16	0.59 ± 0.05	0.89
chloroform	9.3	293 ± 7	148 ± 14	0.51 ± 0.05	0.95
acetone	9.9	281 ± 7	163 ± 16	0.58 ± 0.05	0.91
dioxane	10.0	277 ± 6	193 ± 19	0.70 ± 0.06	0.90
1-octanol	10.3	345 ± 9	164 ± 16	0.48 ± 0.05	1.12
t-butanol	10.4	462 ± 11	156 ± 15	0.34 ± 0.04	1.50
1-butanol	11.4	362 ± 9	163 ± 16	0.45 ± 0.05	1.18
2-propanol	11.5	337 ± 9	153 ± 15	0.45 ± 0.05	1.09
acetonitrile	11.9	196 ± 5	173 ± 17	0.88 ± 0.06	0.64
ethanol	12.7	401 ± 10	159 ± 15	0.40 ± 0.04	1.30
dimethyl sulfoxide	13	259 ± 5	143 ± 14	0.55 ± 0.05	0.84
water	23.4	308 ± 8	156 ± 15	0.51 ± 0.05	1
mineral oil	7.1	precipitates			
hexane	7.3	precipitates			
decane	7.7	precipitates			
tetradecane	7.9	precipitates			
cyclohexane	8.2	precipitates			

Yagi at el. [34] investigated the swelling behavior of PNIPAM gels in different organic solvents. Moreover, they calculated the solubility parameter of the PNIPAM polymer using Equation (1):

(1) $${\left[Q^{-1}\ln\left(Q_{max}∕Q\right)\right]}^{1/2}=\left|a^{1/2}\left(\delta_{sol.}-\delta_{PNIPAM}\right)\right|,$$

where *Q* = (*R*_h_/*R*_h(H_2_O)_)³, *Q*_max_ = (*R*_h(t-butanol)_/*R*_h(H_2_O)_)³— maximum swelling ratio, *δ*_sol._—solubility parameter of the solvent, *δ*_PNIPAM_—solubility parameter of PNIPAM, and *a*—constant.

**Figure 1:**
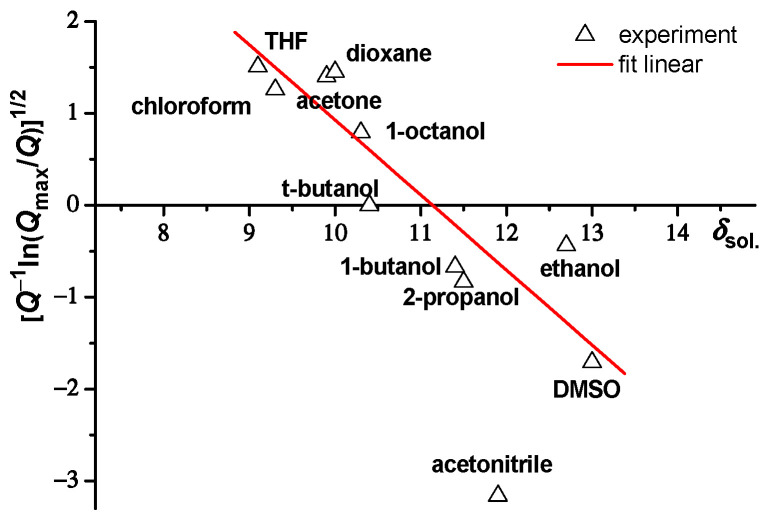
Dependence of ${\left[Q^{-1}ln\left(Q_{max}∕Q\right)\right]}^{1/2}$ on the solubility parameter *δ*_sol._ (THF—tetrahydrofuran, DMSO—dimethyl sulfoxide).

We generated a plot according to [34] (see Figure 1). The solubility parameter of the PNIPAM gel was calculated from the plot, *δ*_PNIPAM_ = 11.14 (*a* = 0.667). This value for PNIPAM shows good agreement with *δ*_PNIPAM_ = 11.5, which was obtained by Yagi et al. [34], and with *δ*_PNIPAM_ = 11.18, which was calculated by Ahmad [54].

We showed that the swelling behavior of PNIPAM microgels (synthesized via precipitation polymerization) in organic solvents can be described by the Gee theory [55].

As seen in Figure 1, the swelling of PNIPAM microgels in acetonitrile solvent deviated from the general trend (see red line in Figure 1). This may be explained by the fact that acetonitrile is a solvent capable of poor hydrogen bonding interactions, unlike other solvents in this study that are capable of moderate and strong hydrogen bonding [34,35,51].

### 2.2. Investigation of the Water Solution of PNIPAM Microgels Loaded with Sudan III Dye

**Figure 2:**
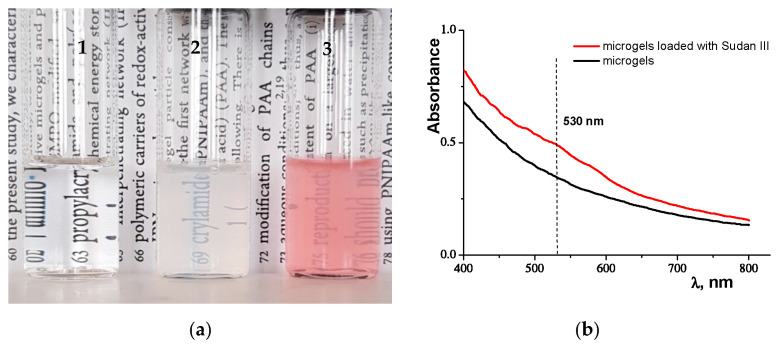
Panel (**a**) shows the flasks filled with: 1—pure water, 2—the water solution of PNIPAM microgels, 3—the water solution of colored PNIPAM microgels. Panel (**b**) shows the UV-visible spectra of the water solution of PNIPAM microgels (black curve) and the water solution of colored PNIPAM microgels (red curve) at 23 °C.

The water dispersions of the microgels loaded with the fat-soluble dye were prepared in a manner described below (see Section 3.4). Figure 2a shows a picture of the aqueous solution of microgels colored with water-insoluble dye Sudan III (3), in comparison to that with pure water (1) and the microgel aqueous solution (2). Below, we will find out why the water solution of Sudan III has a uniform pink color.

#### 2.2.1. UV-Vis Spectrophotometry

It is well known that the color of dyes depends on their ability to absorb light in a visible range of 400–800 nm. We studied the UV-visible absorption spectrum of Sudan III incorporated in microgels and dispersed in pure water. Figure 2b shows that PNIPAM microgels, having swollen in water, did not contain any active group, and there were no absorption peaks in the UV-visible region from 400 nm to 800 nm. However, the aqueous solution of microgels with incorporated Sudan III dye exhibited an absorption peak in the UV-visible region at about 530 nm. This result corresponds to that obtained in a previous paper [56], where it was found that Sudan III dye shows a bathochromic shift in the absorption band with an increase in solvent polarity. Thus, the fat-soluble dye can be homogeneously dispersed in the water solution. The dye molecules are adsorbed onto the polymer chains of the microgels.

It should be noted that the Sudan III used in our experiments is completely insoluble in water. We did not achieve the solubility of the dye in water, even at a concentration of 0.00026 mg/mL.

#### 2.2.2. Ultracentrifugation

**Figure 3:**
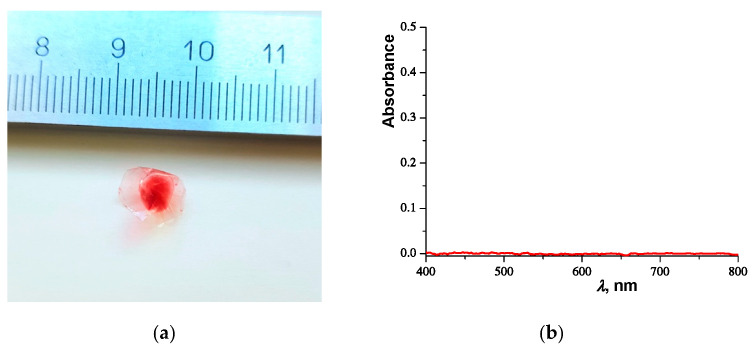
Picture (**a**) shows the dried pellet composed of microgels and Sudan III dye. (**b**) shows the UV-visible spectra of the supernatant formed as a result of the centrifugation of the water solution of the stained microgels.

As a result of centrifugation, we obtained a pink-colored pellet and a colorless supernatant. The pellet was dried at ambient temperature and was a red polymer film (see Figure 3a). The supernatant was investigated with spectrophotometry. The Sudan III dye was not detected in the water solution (see Figure 3b). It can be concluded that Sudan III was adsorbed onto the polymer network of microgels. Similar observations were made for the PNIPAM macrogel (see Figures S1 and S2).

#### 2.2.3. Determination of Sizes of PNIPAM Microgels Loaded with Sudan III Dye

Multi-angle dynamic light scattering was used to obtain the hydrodynamic radius *R*_h_ and radius of gyration *R*_g_ of the microgels. We determined the *R*_h_ and *R*_g_ of standard PNIPAM microgels and microgels with incorporated dye molecules at 20 °C and 45 °C (see Table 2). It was determined that the *R*_h_ of microgels with Sudan III is larger than the size of standard microgels at 20 °C, namely 351 nm vs. 308 nm. The microgels, having absorbed the dye molecules, increased their own size after swelling in water.

The thermo-sensitive property of microgels was investigated. It is shown in Table 2 that the *R*_h_ and *R*_g_ of microgels loaded with the hydrophobic dye at 45 °C were almost the same as those for “standard” microgels. Thus, the microgels with Sudan III dye keep their thermo-sensitivity.

## 3. Materials and Methods

### 3.1. Materials

All materials for microgel synthesis were acquired from Sigma-Aldrich (Munich, Germany) unless stated otherwise. *N*-isopropylacrylamide (NIPAM—monomer,), *N*,*N*’- methylenebisacrylamide (BIS—crosslinking agent), ammonium persulfate (APS—initiator) were used as received. The organic solutions 1-octanol, 1-buthanol, decane, and tetradecane were purchased from Acros organics (Geel, Belgium). Tetrahydrofuran and acetone were received from PanReac AppliChem (Darmstadt, Germany). Chloroform, dioxane, 2-propanol, t-butanol, ethanol, hexane, dimethyl sulfoxide, acetonitrile, and cyclohexane were received from Chimmed (Moscow, Russia). Mineral oil was purchased from VWR (Darmstadt, Germany). The fat-soluble dye Sudan III (analytical standard) was obtained from Sigma-Aldrich (Munich, Germany). Water was purified using a Millipore Milli-Q system.

### 3.2. Synthesis of Microgels

The synthesis of PNIPAM homopolymer microgels was carried out through the classical and most-often-used method of radical thermo-initiated emulsifier-free precipitation polymerization of NIPAM in water in the presence of the crosslinking agent. The NIPAM monomer concentration in the reaction mixture was 1 wt. %; the cross-linker BIS concentration was 1 mol % in terms of the monomer; the initiator APS concentration was 0.07 wt. %. Polymerization was carried out in a glass reactor in an argon atmosphere at a temperature of 80 °C under continuous stirring at a rate of 600 rpm for 24 h. The aqueous dispersion of the synthesized microgels was gradually cooled to room temperature and purified through dialysis (the dialysis bag pore size was 20 kDa) for two weeks. The purified dispersion was dried via lyophilization using a FreeZone 2.5 freeze dryer (Labconco, Kansas City, MO, USA).

### 3.3. Preparation of Solvent Solutions of Microgels

We investigated the dependence of microgel size on the nature of the organic solvent at 23 °C. The following organic solvents were used: pure water, dioxane, chloroform, 1-octanol, 1-butanol, t-butanol, 2-propanol, ethanol, acetone, acetonitrile, dimethyl sulfoxide, tetrahydrofuran, decane, hexane, mineral oil, cyclohexane, and tetradecane. The organic solvent was poured into a glass flask. Some amount of the dry microgels was added to the solvent. Then, the solution was stirred with a magnetic stirrer for two days to obtain a homogenous solution. The concentration of microgels in the solutions was 0.43 mg/mL throughout all experiments.

### 3.4. Loading of Microgels with Sudan III Dye

A solution of water-insoluble Sudan III dye in pure chloroform was prepared. The concentration of Sudan III in chloroform was 6·× 10^−5^ M. Then, 0.0064 g of dry PNIPAM microgels was added to 1 g of colored chloroform. After that, the solution was mixed with a magnetic stirrer for two days at 100 rpm to obtain a homogenous solution. The flask with microgels and colored chloroform was left with a lid open for two days until the chloroform had completely evaporated. To remove all chloroform molecules, the flask was placed in a vacuum drying cabinet at 23 °C for 24 h. As a result of these manipulations, we obtained colored dry microgels. After that, 3 mL of pure water was added and the solution was mixed with a magnetic stirrer to obtain a homogenous microgel solution.

### 3.5. Dynamic and Static Light Scattering Methods

Static light scattering (SLS) and dynamic light scattering (DLS) measurements were performed with a static/dynamic compact goniometer (DLS/SLS-5000, ALV, Langen, Germany). A HeNe laser with a power of 22 mW, emitting a polarized light at *λ* = 633 nm, was used as the incident beam. DLS measurements and SLS intensity functions were taken at 23 °C at scattering angles from 30° to 150° with a step of 10°. The mass concentration of the samples was 0.4 g/L. Distributions over decay time τ were obtained by means of a nonlinear regularized inverse Laplace transformation method (CONTIN) [57]. Apparent self-diffusion coefficients *D* were determined from the angular dependence of the relaxation time τ in accordance with the equation *D* = 1/*τq*^2^, where *q* = (4*πn*/*λ*)sin(*θ*/2) is the wave vector magnitude. The corresponding hydrodynamic radii *R*_h_ presented in Table 1 were calculated from the Stokes–Einstein equation: *R*_h_ = *kT*/6*πηD*, where *k* is Boltzmann’s constant and *η* is the solvent viscosity. The radii of gyration *R*_g_ presented in Table 1 were calculated from the plots of the reciprocal excess intensity normalized to the relative contribution of *I*(*q*) vs. *q*^2^ using the Guinier equation [58]:

(2) $$\ln\left(Kc/R_{\theta }\right)=\ln\left(\frac{1}{M_{w}\exp\left(-\frac{1}{3}R_{g}^{2}q^{2}\right)}+2A_{2}c\right),$$

where $K=4\pi^{2}{\left(dn/dc\right)}^{2}n_{0}{}^{2}/N_{0}\lambda^{4}$, *n*_0_ is the medium refractive index, *N*_0_ is the Avogadro number, d*n*/d*c* is the refractive index increment, *c* is concentration, $R_{\theta }$ is the Rayleigh ratio at the angle *θ*, *M*_w_ is the molecular weight, and *A*_2_ is the 2nd virial coefficient. Solvent viscosity *η* and solvent refractive index *n* table values were taken accordingly to the solvent used in the experiment and incorporated into ALV software.

### 3.6. Spectrophotometry

We used UV-Vis spectrophotometry for the qualitative determination of Sudan III dye in the PNIPAM microgels. A quartz cuvette with a 1 cm path length was used. The UV-visible absorbance spectra in a range of 400–800 nm in steps of 1 nm on an SF-2000 spectrophotometer were obtained. The quartz cuvette and spectrophotometer were purchased from OKB-Spectr (St. Petersburg, Russia). Microgel concentrations in water were 2.1 mg/mL. The concentration of Sudan III in water solution was 0.0046 mg/mL.

### 3.7. Ultracentrifugation of Microgel Water Solution

Ten milliliters of the solution of microgels loaded with Sudan III was placed in a centrifuge tube and subjected to centrifugation at 14,000 rpm for 10 min to separate the solution into fractions. The laboratory centrifuge PE-6926 was received from Ecohim (St. Petersburg, Russia). The pellet was removed from the tube and the supernatant was centrifuged again. The newly separated pellet was removed and collected. The twice-separated supernatant was investigated via UV-Vis spectrophotometry. Microgel concentrations in water were 2.1 mg/mL. The concentration of Sudan III in the water solution was 0.0046 mg/mL.

## 4. Conclusions

In this research, we studied, in detail, the behavior of common PNIPAM microgels, obtained via surfactant-free precipitation polymerization, in a number of organic solvents. We showed that many of the selected solvents serve as good ones for PNIPAM microgels, and the size and architecture of the microgels depend on the solvents chosen. It was demonstrated that the Gee theory can be applied to describe the solubility of PNIPAM microgels, synthesized through precipitation polymerization, in organic solvents. Moreover, the solubility parameter of PNIPAM was obtained. We successfully encapsulated fat-soluble Sudan III dye in PNIPAM microgels and prepared the aqueous dispersion of such composite-colored microparticles. It was shown that the dye molecules adsorb to the polymer network of the PNIPAM microgels. In perspective, such microgel-dye composites could be used as coloring agents that are easily removable from water.

## Figures and Tables

**Table 2:** Characteristics of the sizes of PNIPAM microgels loaded with Sudan III dye in pure water at 20 °C and 45 °C.

Microgels	*R*_h_, nm	*R*_g_, nm	*R*_g_/*R*_h_
“Standard” at 20 °C	308 ± 9	156 ± 15	0.51 ± 0.03
Loaded with Sudan III at 20 °C	351 ± 10	141 ± 14	0.40 ± 0.02
“Standard” at 45 °C	114 ± 5	90 ± 9	0.79 ± 0.06
Loaded with Sudan III at 45 °C	119 ± 5	90 ± 9	0.76 ± 0.06

## Data Availability

All data generated or analyzed during this study are available upon request from the author.

## Supplementary Materials

The following supporting information can be downloaded at: https://www.mdpi.com/article/10.3390/molecules27238549/s1, Synthesis of PNIPAM macrogel; Visual observation of Sudan III behavior inside the macrogel; Figure S1: Pictures of dry colored PNIPAM gel and colored PNIPAM hydrogel having swelled in water; Figure S2: UV-visible spectrum of the outer water solution in which the colored PNIPAM hydrogel was immersed; Figure S3: Normalized intensity correlation functions of PNIPAM microgel solution for different solvents; Determination of the radius of gyration; Figure S4: The angular dependencies of the relaxation time of PNIPAM microgel solutions.

## Sample Availability

Samples of the compounds are available from the authors.
