# Supplementary material for: Behavior of PNIPAM Microgels in Different Organic Solvents
Source: Molecules. 2022 Dec 5;27(23):8549. doi: 10.3390/molecules27238549 (PMC9737493; doi:10.3390/molecules27238549)
Supplement: Supplementary file 1 [file molecules-27-08549-s001.zip › molecules-2035266-supplementary.pdf]

molecules

MDPI

Article

Supporting Information: Behavior of PNIPAM Microgels in Different Organic Solvents

Galina A. Komarova [1], Elena Yu. Kozhunova [1,2,*] and Igor I. Potemkin [1,2,*]

[1] Physics Department, Lomonosov Moscow State University, Leninskie Gory 1-2, Moscow 119991, Russia
[2] A.N. Nesmeyanov Institute of Organoelement Compounds, Russian Academy of Sciences, Vavilova St. 28, Moscow 119991, Russia
* Correspondence: kozhunova@polly.phys.msu.ru (E.Y.K.) and igor@polly.phys.msu.ru (I.I.P.)

1. Synthesis of PNIPAM Macrogel

A film of PNIPAM hydrogel (macrogel) was synthesized by a free-radical polymerization of NIPAM in the presence of BIS as a crosslinker. 0.45 g of NIPAM and 0.0061 g of BIS were dissolved in 4.5 mL of deionized water using a magnetic stirrer. The solution was treated by bubbling of nitrogen for 1 h. Polymerization was initiated after adding 77 µl of 10 wt.% water solution of APS and 4.5 µl of TEMED in monomers' solution. Polymerization proceeded for two days at room temperature in a hermetically sealed cell. After synthesis, PNIPAM hydrogel was washed in deionized water for several days to purify from unreacted chemicals. A thickness of the hydrogel film was 1.3 mm. The thickness of the hydrogel film was measured using calipers.

2. Visual Observation of Sudan III Behavior inside the Macrogel

The piece of PNIPAM macrogel was colored in the same way as microgels. After that, the colored macrogel was placed in pure water, and a behavior of the dye inside polymer was observed. Figure S1(a) presents the piece of the gel after vacuum drying. The piece was placed in pure water where it began to swell. Next day the picture of swollen PNIPAM hydrogel was taken (see Figure S1(b)). It is clearly seen that the dye is distributed inside the macrogel. Also we did not observe a realese of Sudan III dye from hydrogel in water. UV-visible spectrum of the outer water solution shows that it is free of dye (see Figure S2).

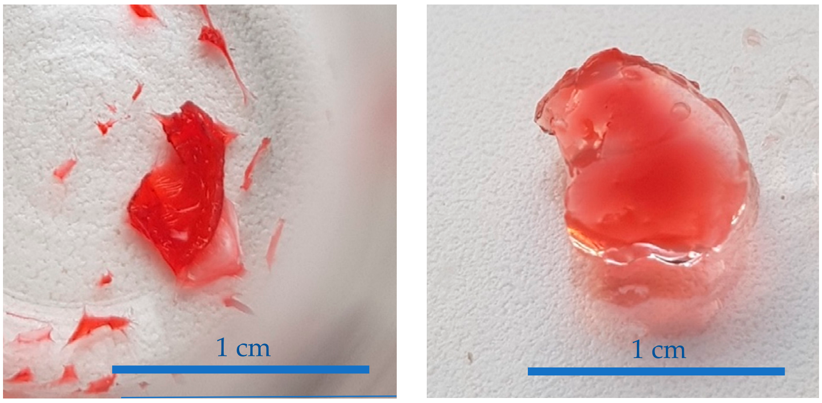

(**a**) (**b**)

Figure S1. Picture (**a**) shows the pieces of the dry colored PNIPAM gel, picture (**b**) shows colored PNIPAM hydrogel having swelled in water at room temperature for 1 day.

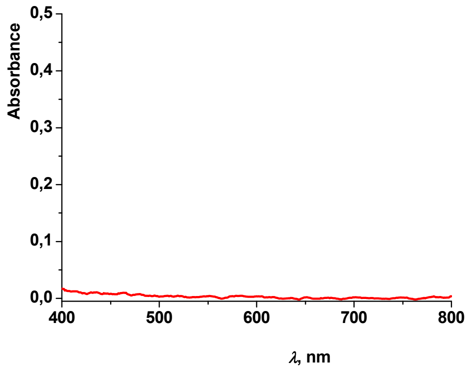

Figure S2. UV-visible spectrum of the outer water solution where colored PNIPAM hydrogel was immersed.

3. Intensity Correlation Function

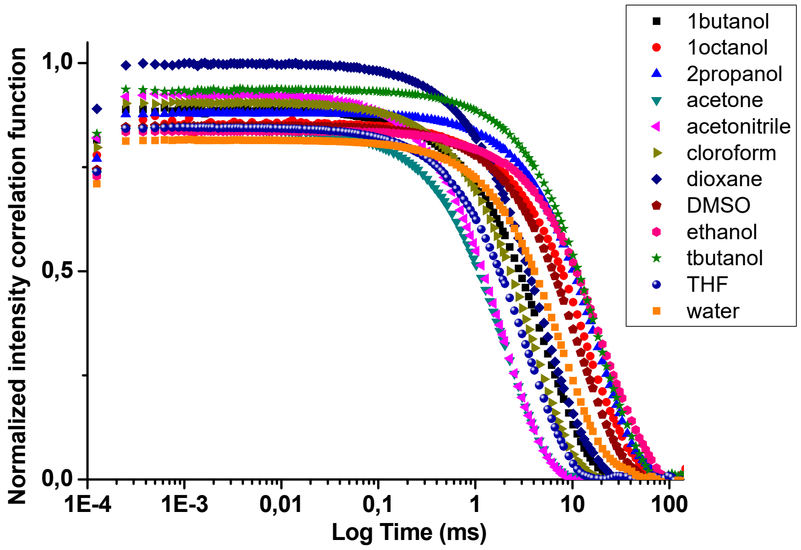

Figure S3. Normalized intensity correlation functions of PNIPAM microgel solution for different solvents measured by dynamic light scattering: 1-butanol, 1-octanol, 2-propanol, acetone, acetonitrile, cloroform, dioxane, dimethyl sulfoxide (DMSO), ethanol, t-butanol, tetrahydrofuran (THF) and water. The scattering angle was 40°.

4. Determination of the Radius of Gyration

The radius of gyration R_g was calculated according to the Guinier [Light Scattering from Polymer Solutions, Ed. by M. B. Huglin (Academic Press, London; New York, 1972)] relations from angular dependences of scattered light intensity. We did not calculate the molecular weight of the microgels in this research; it was done in a separate work using Guinier method. The full package of these dependencies can be requested from the authors.

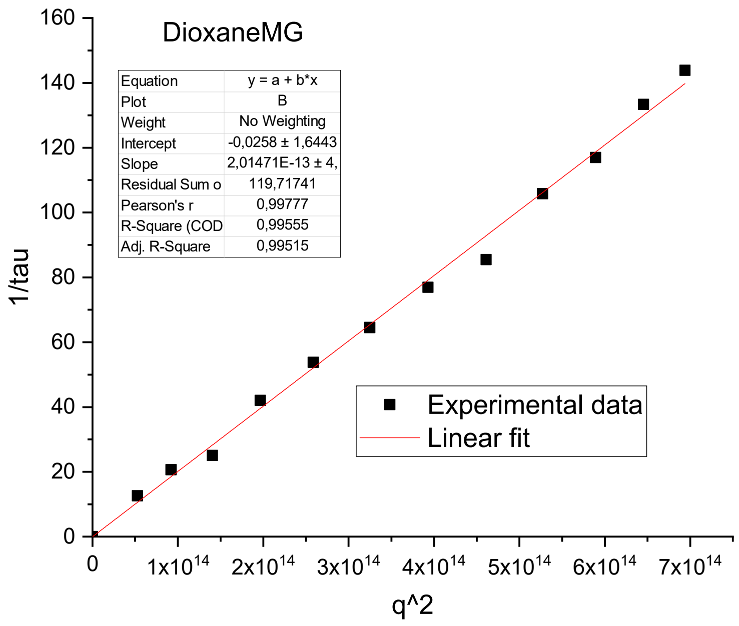
DioxaneMG
Equation y = a + b*x
Plot B
Weight No Weighting
Intercept -0,0258 ± 1,6443
Slope 2,01471E-13 ± 4,
Residual Sum o 119,71741
Pearson's r 0,99777
R-Square (COD 0,99555
Adj. R-Square 0,99515
Experimental data
Linear fit
1/tau
160
140
120
100
80
60
40
20
0
1x10^14
2x10^14
3x10^14
4x10^14
5x10^14
6x10^14
7x10^14
q^2

(**a**)

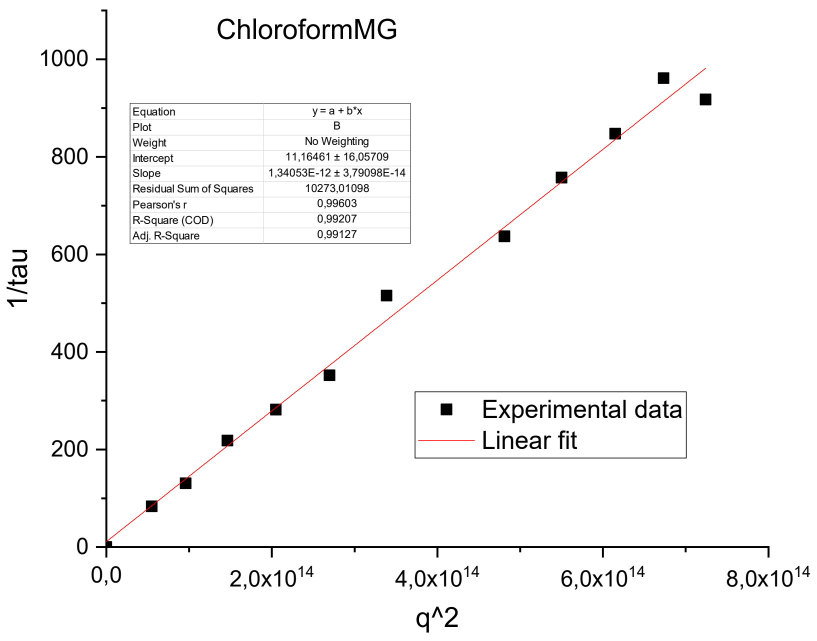
ChloroformMG
Equation y = a + b*x
Plot B
Weight No Weighting
Intercept 11,16461 ± 16,05709
Slope 1,34053E-12 ± 3,79098E-14
Residual Sum of Squares 10273,01098
Pearson's r 0,99603
R-Square (COD) 0,99207
Adj. R-Square 0,99127
Experimental data
Linear fit
1/tau
1000
800
600
400
200
0
0,0
2,0x10^14
4,0x10^14
6,0x10^14
8,0x10^14
q^2

(**b**)

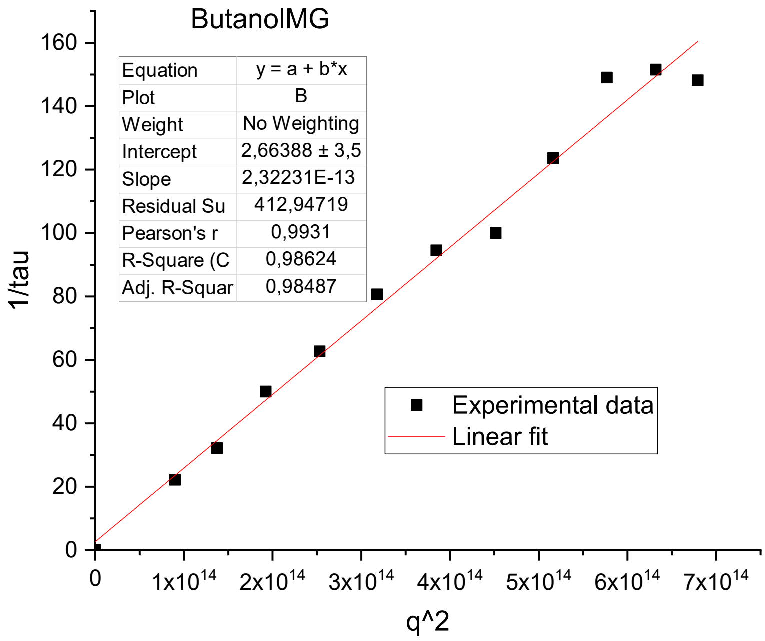

(**c**)

Figure S4. The angular dependencies of the relaxation time of PNIPAM microgel solutions measured by dynamic light scattering: (**a**) dioxane, (**b**) chloroform, (**c**) 1-butanol.
